# Understanding the Relationship between Financial Literacy and Chinese Rural Households’ Entrepreneurship from the Perspective of Credit Constraints and Risk Preference

**DOI:** 10.3390/ijerph20064981

**Published:** 2023-03-11

**Authors:** Silin Liu, Jia He, Dingde Xu

**Affiliations:** 1 Committee of the Communist Youth League, Sichuan Agricultural University, Chengdu 611130, China; 2 College of Management, Sichuan Agricultural University, Chengdu 611130, China; 3 Sichuan Center for Rural Development Research, Sichuan Agricultural University, Chengdu 611130, China

**Keywords:** financial literacy, entrepreneurship, rural areas, credit constraints, risk preference

## Abstract

Farmers’ entrepreneurship is an important factor in promoting rural economic growth, yet few studies have systematically discussed the impact of financial literacy. Using the 2021 China Land Economic Survey data, this study analyzes the relationship between financial literacy and Chinese rural households’ entrepreneurship from the perspective of credit constraints and risk preferences by the IV-probit, stepwise regression, and moderating effects methods. This study finds that: (1) the financial literacy of Chinese farmers is low, with only 11.2% of the sample households starting businesses; and (2) Financial literacy can promote rural households’ entrepreneurship. After introducing an instrument variable to control endogenous problems, the positive correlation is still significant; (3) financial literacy effectively alleviates the traditional credit constraints of farmers, thereby promoting entrepreneurship; (4) risk preference weakens the positive impact of financial literacy on rural households’ entrepreneurship. This study provides a reference for optimizing entrepreneurship policies.

## 1. Introduction

The world today is at the juncture of unprecedented changes and unprecedented outbreaks. The epidemic of COVID-19 has spread globally, the international political pattern is unstable, and most economic and social activities have been destructively affected [1]. Entrepreneurship is regarded as an important driving force for economic development. Because entrepreneurial activities can not only increase income but also provide employment opportunities [2,3]. Global Entrepreneurship Monitor (GEM) data shows that countries with more entrepreneurs and more active entrepreneurship have higher economic growth rates [4]. Especially in developing countries with large rural populations, farmers’ entrepreneurship is an important way to change the agricultural production pattern and promote rural economic growth and social development [5,6]. Therefore, the driving factors of entrepreneurship have gradually become the research focus of the academic community.

Previous studies have analyzed the influencing factors of entrepreneurial behavior and performance from both internal and external aspects. Among them, the internal factors mainly include the personal characteristics and social capital of entrepreneurs [7,8,9,10]. External factors mainly refer to the entrepreneurial environments, such as background factors such as policy system, economy, technology, and culture [11,12,13]. In addition, financial literacy is also closely related to entrepreneurship [14,15,16]. Financial literacy is an important symbol of entrepreneurial talent. It measures the ability of individuals to master financial concepts and manage and allocate financial resources [17]. In the process of entrepreneurship, entrepreneurs need to identify entrepreneurial opportunities, raise entrepreneurial funds, manage entrepreneurial resources, and run enterprises [2]. These links inevitably involve financial problems. Therefore, it can be said that good financial literacy is an important condition for entrepreneurship and entrepreneurial success [16]. However, research on financial literacy and entrepreneurship usually focuses on young people or urban residents and pays little attention to rural households. It should be noted that rural residents face more restrictive factors in entrepreneurship compared to urban residents [15]. In particular, rural residents of developing countries have low levels of education and lack the natural resources needed for entrepreneurship. So, it is harder to start a business [6]. In the limited research focusing on rural households’ financial literacy and entrepreneurship in developing countries, developing countries, endogenous issues and underlying mechanisms have been neglected [12,13]. Will financial literacy affect the entrepreneurial behavior of rural residents? If so, what is the internal transmission mechanism? The answers to these questions are of great significance to rural revitalization and poverty eradication. This paper chooses China as the background to study the relationship between rural households’ financial literacy and entrepreneurship. First of all, China is the largest developing country in the world, with a large rural population. In 2021, China currently has 498.35 million rural people. Secondly, the Chinese government particularly encourages rural households to start businesses. In February 2015, China’s State Council for the first time published an official document on farmers’ entrepreneurship as an important way to accelerate rural development. Since then, the Chinese government has continued to introduce policies such as financial subsidies, loan incentives, and tax exemptions, hoping to improve rural households’ enthusiasm for entrepreneurship [18]. Especially after the victory of China’s fight against poverty, farmers’ entrepreneurship is regarded as an important method to stabilize the effect of poverty alleviation and prevent poverty eradication and rural revitalization [13]. Finally, with the development of China’s society, residents’ financial literacy has improved significantly. According to the Investigation and Analysis Report on Consumer Financial Literacy 2021 of the People’s Bank of China, the financial literacy level of Chinese residents is above average in the world. However, it should be noted that most of the respondents are urban residents. Whether farmers’ financial literacy has been significantly improved remains to be further tested.

Based on the data of 2278 rural households in Jiangsu Province, China, this study uses the Probit model, IV probit model, stepwise regression, and moderating effect test methods to empirically analyze the relationship and internal mechanism between financial literacy and rural households’ entrepreneurship with a hope to provide a reference for optimizing entrepreneurship policies. Compared with the existing research, the possible marginal contribution of this paper lies in the following. First, from the perspective of human capital—financial literacy, it analyzes the path to promote rural households’ entrepreneurship, which enriches the research on the antecedents of entrepreneurship. Second, the objective credit constraints and subjective risk preferences are included in the analysis framework, and the mediating effect and moderating effect models are constructed. It empirically discusses the mediating role of easing credit constraints and the moderating role of risk preference and deepens the understanding of the relationship between financial literacy and rural households’ entrepreneurship in different contexts. Third, this paper utilizes 2278 micro survey data samples, controls for endogenous problems, and pays attention to the financial literacy and entrepreneurial behavior of rural households in developing countries, a special group.

## 2. Theoretical Framework

### 2.1. Financial Literacy and Rural Households’ Entrepreneurship

In order to play a role in economic prosperity, entrepreneurship must have sufficient human capital [15]. Academics generally believe that financial literacy, as a special human capital, plays a decisive role in family finance and financial decision-making [19,20,21]. Financial literacy was originally proposed by Noctor and Stradling and defined as the ability of individuals to allocate and manage funds, which enables them to accurately judge the future form and make correct decisions. Subsequent studies defined financial literacy from different perspectives, such as knowledge, application, and awareness [22,23]. Financial literacy reflects the entrepreneurial ability accumulated by individuals in the financial or economic fields and becomes one of the antecedents of entrepreneurship [24]. On the one hand, farmers with rich financial knowledge and good financial literacy can judge the opportunity cost and marginal income in entrepreneurial activities, collect and analyze market information, and explore entrepreneurial opportunities [10]. On the other hand, good financial literacy can help farmers reasonably plan future expenses and income, improve the management level of investment decisions, and ensure proper financial management of entrepreneurial projects [13]. In addition, starting a business requires activities such as bookkeeping and tax payment. Farmers with good financial literacy can undertake these tasks by themselves, avoiding hiring accountants and saving startup costs. Based on this, hypothesis 1 is proposed.

**H1.**  *Financial literacy significantly promotes rural households’ entrepreneurship*.

### 2.2. The Mediating Role of Easing Credit Constraints

Improving financial literacy will help ease credit constraints and then promote entrepreneurship. Entrepreneurship requires money investment, and there is a minimum capital threshold. In addition to original self-owned capital and borrowing money from relatives and friends, borrowing in the financial market is another way to raise funds. However, China’s credit distribution policy tends to “attach importance to cities and neglect rural areas”, which means that rural households have fewer financial resources and more strict conditions for obtaining financial support [12]. Financial literacy can alleviate the inhibition of this policy tendency on rural households’ entrepreneurship. First of all, cognition is the premise of practice, which shows that the premise of rural households’ borrowing is to understand the information about loans. Higher financial literacy means that individuals can better understand the borrowing process, interest rate and interest, inflation, and the time value of money [19]. Secondly, financial literacy reflects personal ability and credit level. Individuals with high financial literacy are more likely to pass the qualification examination of financial institutions and obtain loans more easily [25]. Huston (2012) [24] found that compared with other consumers, consumers with high financial literacy have lower credit card interest rates and lower mortgage costs. Third, rural households with high financial literacy can correctly allocate their limited resources and may also participate in financial activities such as stocks and funds to promote family wealth accumulation [13]. This represents the ability to pay interest. It can be seen that financial literacy can ease rural households’ credit constraints [26].

Many studies show that credit constraints limit the rate of entrepreneurship and the size of enterprises [12]. To this end, many countries have relaxed bank regulations to allow more people to obtain financing to improve entrepreneurship [27]. China has proposed an inclusive financial policy, in which a specific goal is to promote entrepreneurial activities and thus eliminate poverty [12]. Credit support is conducive to enhancing individual capital endowment, providing financing for individuals lacking startup funds, and thus improving their entrepreneurial enthusiasm. This conclusion is also applicable to rural households. That is, access to financing is positively correlated with entrepreneur decisions [28]. Furthermore, credit can be divided into the following three types. The first type is traditional credit provided by financial institutions such as banks [26], the second is digital credit provided by financial institutions such as banks [10], and the third is online credit provided by online platforms such as WeChat and Alipay [29]. Based on this, hypothesis two is proposed.

**H2.**  *Financial literacy promotes rural households’ entrepreneurship by easing credit constraints*.

### 2.3. Moderating Role of Risk Preferences

Risk preference may play a moderating role in the positive correlation between financial literacy and rural households’ entrepreneurship. Risk preference refers to an individual’s mental state or reflective strategy for future uncertainty and is a leading factor in perceiving decision-making situations and making behavioral decisions [17]. Risk preference shows a positive attitude in the face of future uncertainty, while risk-averse people are more cautious in taking conservative actions [30]. Entrepreneurship is an activity with both risks and benefits. Previous studies have shown that individual risk preference will affect entrepreneurial decision-making [31]. Generally speaking, the more risk preference, the stronger the motivation to start a business [32]. However, most rural households are risk averse because of their small capital and weak anti-risk ability. Rural households usually choose small-scale, low technology threshold labor-intensive industries to start their businesses. Rural entrepreneurs are also faced with low success rates, low income, and other practical problems [18]. The research speculates that financial literacy has a stronger positive impact on the entrepreneurial decisions of risk-averse people, while risk-preference people are more likely to start their businesses even if they do not have good financial literacy, so the impact of financial literacy on them is weakened. Based on this, hypothesis 3 is proposed.

**H3.**  *Risk preference plays a moderating role in financial literacy and rural households’ entrepreneurship*.

**Figure 1:**
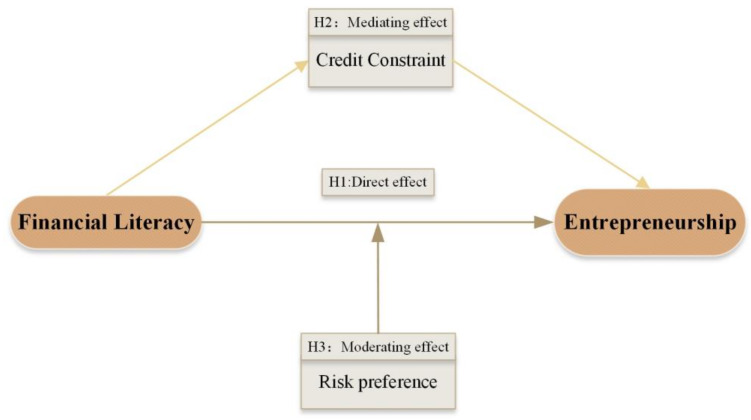
Theoretical Framework.

Based on the above analysis, the research constructs a theoretical framework of financial literacy and rural households’ entrepreneurship from the perspective of credit constraints and risk preference in Figure 1.

## 3. Research Design

### 3.1. Research Data

The data of this study is from the China Land Economic Survey (CLES) of Nanjing Agricultural University. The survey will be carried out in Jiangsu, China, in 2021. Jiangsu is located in the middle of the east coast of mainland China, spanning 30°45′–35°08′ N and 116°21′–121°56′ E. In 2021, Jiangsu’s GDP will rank second in China, and it will be one of the most economically developed provinces in China. Its land area is about 107,200 square kilometers, and its permanent population is about 85.054 million. The Jiangsu government has actively introduced policies to promote rural households’ entrepreneurship by cultivating new agricultural business entities, developing rural e-commerce, guiding the integration of primary, secondary, and tertiary industries, and strengthening characteristic industries. The survey mainly includes basic family information, family production and operation, and capital flows. PPS sampling and simple random sampling were adopted in the survey. According to the sampling process of “city-county-township-village-farmer”, 48 villages were finally selected. Combined with the content of this study, 2278 sample data were obtained after removing missing values and outliers.

### 3.2. Variable Definition and Description

#### 3.2.1. Independent Variable

The independent variable of this study is financial literacy, which has not been uniformly defined by the academic community. Refer to Huston [24], Zhao and Li [7], and Lusardi and Mitchell [19] to judge their financial literacy from both financial knowledge and financial attitude. This study is consistent with Lusardi and Mitchell [19], which believes that the answers “I don’t know” and wrong answers represent two different financial literacy. Therefore, in addition to financial information concerns, each question is measured from two aspects: whether the answer is correct or not. See Table 1 for specific financial literacy indicators. However, after analysis, it is found that the indicator of “financial information attention” is independent, and the variance contribution rate is only 0.9%. Therefore, factor analysis will be conducted again after removing this indicator. The results show that the factor loads of all indexes are greater than 0.5. The *p* value of Bartlett’s spherical test is 0.000, and the KMO result is 0.677, greater than 0.5, indicating that the sample is suitable for factor analysis [33]. After rotation, three common factors with eigenvalues greater than 1 were obtained, and the cumulative variance contribution rate was 78.56%. As shown in Table 1, farmers’ financial literacy is low. Only 20% of farmers can correctly calculate bank deposit interest, and only 5.1% of farmers can understand inflation and the time value of money. Only 8.9% of farmers understand the risk difference between stocks and funds, and 12% of farmers pay attention to economic and financial information. Notably, 39.7% of farmers understand that a venture portfolio can reduce risk. The venture portfolio question, which was far more participatory than the other questions, was answered by 57% of farmers. This may be because the problem is easy to understand. After all, it fits in with the actual production situation of farmers.

#### 3.2.2. Dependent Variable

The dependent variable of this study is entrepreneurship. Through “Does your family start a business?” To measure, if the farmer answers yes, the value is assigned as 1; otherwise, the value is assigned as 0. It can be seen from Table 2 that only 11.2% of rural households start businesses.

#### 3.2.3. Control Variables

Referring to the research of Cai et al. [18] and Jiang et al. [8], control variables are selected from three aspects: individual characteristics, family characteristics, and village characteristics. Among them, individual characteristics include the age, gender, health status, education level of the household head, and the respondent’s perception of entrepreneurial risk; Family characteristics include the number of family members, whether there are cadres, whether there are Party members, the number of relatives who start businesses and whether they have experience of failure in entrepreneurship; Village features include distance to the nearest bank, entrepreneurship training, and financial lectures. It can be seen from Table 2 that most of the heads of households are male elderly people who are healthy and have an education level of about primary and middle school. There are about three permanent residents in the sample families. The number of cadre families and party member families is relatively small. Few relatives and friends start businesses. The nearest bank is about 3 km away. In 2020, the government held one entrepreneurship training and one financial knowledge lecture on average.

#### 3.2.4. Instrumental Variable

Entrepreneurship is a complex investment activity. In this process, entrepreneurs may improve their understanding of economic and financial knowledge and improve their financial literacy in capital allocation and management. Thus, this reverse causality may overestimate the effect of financial literacy. In order to avoid endogenous problems, the research needs to introduce an instrumental variable. A large number of studies on farmers’ behavioral decision-making have taken village-level data as tool variables [7,34]. On the one hand, the village-level financial literacy level has no direct relationship with the entrepreneurship of the sample rural households, meeting the exclusive requirements of instrumental variables. On the other hand, residents of the same village often communicate with each other, and the training and lectures organized by the government are mostly village-based. The financial literacy level of the sample rural households will be affected by the financial literacy level of the village level and other rural households, meeting the requirements of tool variable correlation. This study selects “the average financial literacy of others in the same village except himself” as the instrumental variable. The calculation formula is: $IV_{i|v}=\frac{\Sigma_{1}^{n}FL_{i}-FL_{i}}{n-1}$. $FL_{i}$ which refers to the financial literacy of farmers, and where n means the number of interviewees in the village.

#### 3.2.5. Mediating Variable

According to the theoretical framework, easing credit constraints is the mediating mechanism of financial literacy affecting entrepreneurship. Credit constraints can be divided into three types: traditional credit constraints, digital credit constraints, and online credit constraints. With reference to the research of Jappelli [21], Lee and Sawada [30], and others, the traditional credit constraint is measured by “Do you know about traditional credit operations introduced by formal financial institutions such as banks?”; digital credit constraint is measured by “Are you aware of digital credit operations introduced by formal financial institutions such as banks?”; The online credit constraint is measured by “Do you know about online credit services launched by online platforms such as Alipay and WeChat?”. If the respondent answers “Did not apply” or “applied but was not granted credit”, they are considered to be credit constrained. This means that the variable is assigned 1, while the opposite is assigned 0.

#### 3.2.6. Moderating Variable

According to the analysis framework, the impact of financial literacy on rural households’ entrepreneurship will be regulated by risk preference. Define rural households’ risk preferences by asking them about their investment preferences for low, medium, and high risks. Table 2 shows that most of the sample rural households are risk averse and tend to choose investment projects with low risk, low income, and low loss.

Table 2 reports the definitions of all variables and simple descriptive statistical results.

### 3.3. Research Methods

#### 3.3.1. Benchmark Method

The dependent variable is 0–1 variable. Therefore, this study set the following binary Probit model to empirically test the relationship between financial literacy and rural households’ entrepreneurship.

(1) $$Y_{i}=\beta_{0}+\beta_{1}X_{i}+\beta_{2}Control_{i}+\varepsilon_{i}$$

In Formula (1), $Y_{i}$ means that entrepreneurship; $X_{i}$ means financial literacy; $Control_{i}$ is a group of control variables; $\beta_{0}$ is a constant term; $\varepsilon_{i}$ is a random disturbance term; and $\beta_{1}$ is the regression coefficient.

#### 3.3.2. Endogenous Treatment

In order to solve the endogenous problem, the following IV-Probit model is set:

(2) $$X_{i}=\beta_{0}+\beta_{1}IV_{i|v}+\beta_{2}Control_{i}+\varepsilon_{i}$$

(3) $$Y_{i}=\beta_{0}+\beta_{1}FL_{i}+\beta_{2}Control_{i}+\varepsilon_{i}$$

Formula (2) is the first stage regression of IV Probit to test the correlation between village-level financial literacy and farmers’ financial literacy. Wherein, $IV_{i|v}$ refers to village-level financial literacy. Formula (3) is the second stage regression of IV-Probit to test the relationship between financial literacy and rural households’ entrepreneurship. Among them, $FL_{i}$ is $X_{i}$, obtained by regression in the first stage.

#### 3.3.3. Test Method of Mediating Effect and Moderating Effect

This study tests the impact mechanism of financial literacy on rural households’ entrepreneurship through stepwise regression. The stepwise regression method is divided into three steps. The first step tests the relationship between financial literacy and rural households’ entrepreneurship, which is the same as Formula (1), so it will not be repeated. The second step is to test the relationship between financial literacy and mediating variable. The third step includes mediating variable into the regression model of the first step. The specific model settings of the second and third steps are as follows:

(4) $$M_{i}=\beta_{0}+\beta_{1}X_{i}+\beta_{2}Control_{i}+\varepsilon_{i}$$

(5) $$Y_{i}=\beta_{0}+\beta_{1}X_{i}+\beta_{2}M_{i}+\beta_{3}Control_{i}+\varepsilon_{i}$$

If $\beta_{1}$ is significant in (1), $\beta_{1}$ is significant in (4), and $\beta_{2}$ is significant in (5). This shows that the mediation effect has been verified.

In order to test the moderating effect of risk preference, this study introduced the cross-multiplying term of financial literacy and risk preference in Formula (3). The specific model settings are as follows:

(6) $$Y_{i}=\beta_{0}+\beta_{1}FL_{i}+\beta_{2}RP_{i}*FL_{i}+\beta_{3}Control_{i}+\varepsilon_{i}$$

All the above models are implemented through stata17.0.

## 4. Results

### 4.1. Correlation between Financial Literacy and Rural Households’ Entrepreneurship

Table 3 reports the regression results of financial literacy and rural households’ entrepreneurship. Among them, Model 1 and Model 2 are the results obtained based on Probit, and Model 3 and Model 4 are the results obtained based on IV-Probit considering endogenous problems. The chi-square test values of all models are significant at the 1% statistical level, indicating that the model fitting effect is good.

As shown in Table 3, no matter whether control variables are considered or endogenous issues are considered, financial literacy is always positively correlated with rural households’ entrepreneurship. Hypothesis 1 is validated. The higher their financial literacy, the more likely they are to start businesses. The first-stage estimation results of model 3 and model 4 show that the village-level financial literacy rate has a significant positive impact on farmers’ financial literacy. This shows that the higher the village-level financial literacy rate, the better the farmers’ financial literacy. The F test values of the first stage regression were 201.42 and 35, respectively, which were significantly bigger than 10. This result rejects the original hypothesis of weak instrumental variables. In addition, endogenous Wald χ2 is Significant, indicating that there is a systematic difference between the Porbit model and the IV-Probit model, and the selection of instrumental variables in this study is reasonable.

In addition, the significance and significant direction of control variables in Model 2 and Model 4 are consistent. Specifically, the age of the household head in individual characteristics is negatively correlated with entrepreneurship; However, the health of the household head is positively correlated with entrepreneurship. In the family characteristics, the number of families is positively correlated with entrepreneurship; There is a significant positive correlation between the number of entrepreneurs in relatives and entrepreneurship; Entrepreneurial failure experience is significantly positively correlated with entrepreneurship. In the village characteristics, the distance from the village to the nearest bank is positively correlated with entrepreneurship. These results are consistent with existing studies [7,8,35].

### 4.2. Mediating Role of Credit Constraints

As shown in Table 4, it can be seen from Model 6 that financial literacy significantly reduces traditional credit constraints. Model 7 shows that traditional credit constraints significantly inhibit rural households’ entrepreneurship, which is consistent with the previous hypothesis. The promotion of financial literacy in rural households’ entrepreneurship is partly achieved by easing traditional credit constraints. It can be seen from Model 8 that financial literacy has significantly reduced digital credit constraints. This shows that improving financial literacy can ease the constraints of digital credit. However, the results of Model 9 show that digital credit constraints do not play an intermediary role. It can be seen from Model 10 that financial literacy has significantly reduced online credit constraints. This shows that improving financial literacy can ease online credit constraints. However, the results of Model 9 show that online credit constraints do not play an intermediary role. The possible reason is that digital credit and online credit have not been fully popularized, and rural households have little knowledge of them. However, traditional bank loans have been popularized for many years, and rural households are more inclined to choose traditional credit when starting businesses.

### 4.3. Moderating Role of Risk Preference

**Figure 2:**
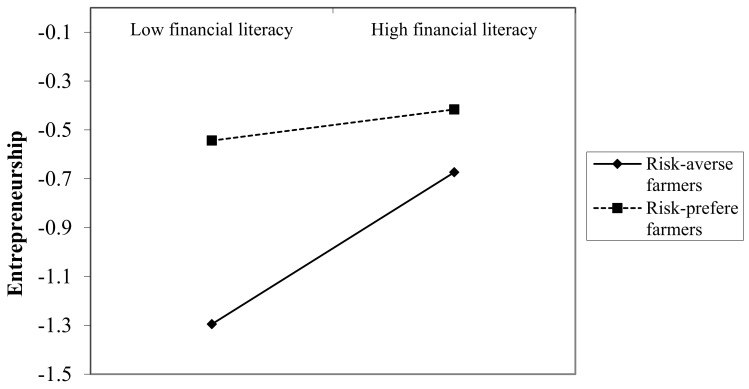
The moderating role of risk preference.

Table 5 and Figure 2 are the tests of the moderating effect of risk preference between financial literacy and rural households’ entrepreneurship. As shown in Table 5, financial literacy is significantly positive, while the cross-term of risk preference and financial literacy are significantly negative, indicating that risk preference weakens the positive impact of financial literacy on rural households’ entrepreneurship. At the same time, there is a significant positive correlation between risk preference and entrepreneurship. As shown in Figure 2, the slope of risk aversion is greater than that of preference risk. This shows that when rural households are risk averse, the positive impact of financial literacy on rural households’ entrepreneurship is more obvious, but with the increase of risk preference, the positive role of financial literacy gradually weakens. In other words, there is an obvious substitution relationship between risk preference and financial literacy in influencing rural households’ entrepreneurship. This may be because risk preference has a greater impact on entrepreneurship. The more risk preference rural households have, the more likely they are to start businesses. Although the farmer does not have complete financial knowledge, he may still start a business if he has a strong preference for risk. It is just that his business is likely to be a low-performing one. By improving financial literacy, risk-averse farmers can better identify entrepreneurial opportunities, raise entrepreneurial funds and have confidence in financial management, so the probability of entrepreneurship is significantly increased.

### 4.4. Robustness Test

In this study, the robustness was tested by replacing the model and key variables. First, the IV-probit model is replaced by Conditional Mixed Process (CMP). The CMP model can introduce an instrumental variable to control endogenous problems and through whether atanhrho_12 is significant to judge whether the results are reliable [34]. Second, this study changed the measurement method of independent variable financial literacy to sum scores. That is, according to the indicators selected above, if the farmers answer correctly or pay attention to financial knowledge, the value will be 1; otherwise, the value will be 0. The scores of the five indicators are added to obtain the new financial literacy variable, and then the regression is repeated.

Table 6 shows the results of the robustness test. No matter what method is used, financial literacy significantly impacts rural households’ entrepreneurship. This shows that the conclusions of this study are robust.

## 5. Discussion

Since entrepreneurship plays an important role in economic growth and social development, the government has been trying to improve people’s entrepreneurial enthusiasm, and the academic community has also paid great attention to it. As a part of human capital, financial literacy affects entrepreneurship. However, the literature usually focuses on young people and urban residents, lacking attention to rural households in developing countries, credit constraints, and risk preferences. In terms of the reality in China and other developing countries, China’s rural financial system is relatively underdeveloped. To promote rural households’ entrepreneurship, the government, on the one hand, relaxed loan policies, such as promoting rural property right asset mortgage loans, entrepreneurial guarantee loans, etc. [12]. On the other hand, small and micro enterprises run by rural households will be given tax incentives [35]. It can be seen that the main measure of the government is to objectively increase money investment. However, improving rural households’ financial literacy has not been given enough attention [13]. Some regions may hold financial knowledge lectures, but due to the times and quality, the improvement of financial literacy is not obvious.

## 6. Conclusions

Using the microdata of 2278 rural households in 2021, this study empirically tested the impact of financial literacy on Chinese rural households’ entrepreneurship and incorporated credit constraints and risk preferences into the analysis framework. The main conclusions are as follows. (1) Improving financial literacy has a positive impact on rural households’ entrepreneurship. This conclusion is still robust after controlling endogenous problems, replacing model methods and key variables. (2) Mechanism analysis found that financial literacy effectively alleviated rural households’ traditional credit constraints, digital credit constraints, and online credit constraints. However, only the traditional credit constraints were alleviated and passed the intermediary mechanism test. (3) Risk preference weakens the positive impact of financial literacy on rural households’ entrepreneurship. (4) The role of financial literacy in promoting rural households’ entrepreneurship is stronger in suburban areas, villages with rural industries, and rural households with low education backgrounds but not in households with a high education background.

Research and suggest several policy suggestions to optimize the entrepreneurship policy. First of all, we can promote entrepreneurship by improving rural households’ financial literacy from the micro level. Specifically, grass-roots governments can cooperate with financial institutions to provide rural households with free training on basic financial knowledge, interpret financial policy information, and provide suggestions on family financial management. Secondly, increase investment in financial education and financial knowledge education in the rural compulsory education stage. Strengthen publicity and guidance, and create a good atmosphere for active learning of financial knowledge. Encourage rural households to learn financial knowledge independently through social networks, the Internet, and other channels. Finally, rural households should deepen their understanding of digital credit and online credit to broaden their lending channels.

This study has some limitations. For example, limited by data, this study only focused on whether to start a business and did not explore the relationship between financial literacy and entrepreneurial performance. However, the establishment of successful enterprises can provide employment and prosper the economy. Whether and how entrepreneurs’ financial literacy can make enterprises more successful remains to be further tested. In addition, this study did not fully reveal the impact mechanism of financial literacy on entrepreneurship but only considered the mitigation of credit constraints.

## Figures and Tables

**Table 1:** Financial literacy measurement index system.

Dimension	Question	Variable	Assignment	Mean	SD
Interest rate	Suppose you have 100 now, and the annual interest rate of the bank is 4%. How much is the total amount of the five-year principal and interest? (A. Less than 120 yuan; B. More than 120 yuan; C. Equal to 120 yuan; D. I don’t know)	Answer the interest rate question	Choose D to be assigned 0, otherwise 1	0.234	0.424
		Answer the interest rate question correctly	Choose C to be assigned 0, otherwise 1	0.200	0.400
Inflation and time value	Suppose you have 100 yuan now, the bank’s annual interest rate is 5%, and the inflation rate is 3% every year. After the 100 yuan is deposited in the bank for a year, what will you buy compared with what you buy now? (A. More; B. Less; C. Same; D. I don’t know)	Answer the inflation and time value question	Choose D to be assigned 0, otherwise 1	0.173	0.378
		Answer the inflation and time value question correctly	Choose A to be assigned 0, otherwise 1	0.051	0.220
Stocks and funds	Do you think buying a stock alone is riskier than buying a stock fund? (A. Yes; B. No; C. I don’t know; D. I haven’t heard of stocks; E. I haven’t heard of funds; F. I haven’t heard of either)	Answer the stocks and funds question	Choose A or B to be assigned 0, otherwise 1	0.111	0.315
		Answer the stocks and funds question correctly	Choose A to be assigned 0, otherwise 1	0.089	0.284
Venture portfolio	Do you think that, in general, planting (operating) multiple crops is less risky than planting (operating) one crop? (A. Yes; B. No; C. I don’t know)	Answer the venture portfolio question	Choose C to be assigned 0, otherwise 1	0.570	0.495
		Answer the venture portfolio question correctly	Choose A to be assigned 0, otherwise 1	0.397	0.489
Financial information attention	Do you usually pay attention to economic and financial information?	Financial information attention	Very concerned, very concerned, general = 1; Little or no attention = 0	0.120	0.325

**Table 2:** Variable Definition Table.

Variable		Variable Definition	Mean	SD
Dependent variable	Entrepreneurship	Does your family start a business? (1 = Yes, 0 = No)	0.112	0.315
Independent variable	Financial literacy	Calculated by factor analysis, the questions and answers are shown in Table 1	0	0.585
Control variables—individual characteristics	Age of household head	Unit: Year old	63.35	10.52
	Gender of household head	1 = MaN; 0 = Female	0.925	0.264
	Health status of household head	Self-identified health status (1 = incapacity; 2 = poor; 3 = medium; 4 = good; 5 = excellent)	4.013	1.092
	Education of household head	Unit: year	7.293	3.649
	Risk perception	What do you think about the risk of failure faced by entrepreneurship? (1 = unknown; 2 = relatively small; 3 = average; 4 = relatively big; 5 = very big)	3.566	1.477
Control variable—family characteristics	Number of families	How many people live in your family (for 6 months or more in a year)?	3.057	1.600
	Cadre	Are there any cadres in your family? (1 = Yes, 0 = No)	0.157	0.364
	Party member	Are there any Party members in your family? (1 = Yes, 0 = No)	0.309	0.462
	Number of entrepreneurs among relatives	How many of your relatives and friends start businesses?	0.560	2.413
	Entrepreneurship failure experience	Has your family experienced any entrepreneurial failure since 2015? (1 = Yes, 0 = No)	0.0160	0.124
Control variable—village characteristics	Distance from the village to the nearest bank	Distance from the village committee to the nearest bank (available for counter business) (km)	3.344	2.942
	Entrepreneurship training	Number of entrepreneurship training organized by township governments in 2020 (times)	1.887	1.347
	Financial Lectures	How many lectures on financial knowledge have been held in the village in 2020? (times)	1.147	1.192
Instrumental variables	Financial literacy level at a village level	Average financial literacy of others in the same village except himself	−0.002	0.206
Mediating variable	Traditional credit constraints	Do you understand the traditional credit business launched by banks and other formal financial institutions? (No application or application but no credit = 1; others = 0)	0.957	0.204
	Digital credit constraints	Do you understand the digital credit business launched by banks and other formal financial institutions? (No application or application but no credit = 1; others = 0)	0.995	0.072
	Bank credit constraints	Do you understand the online credit business launched by Alipay, WeChat, and other network platforms? (No application or application but no credit = 1; others = 0)	0.983	0.128
Moderating variable	Risk preference	Which of the following investments would you prefer? (1 = investment with low risk, low risk, low return, and low loss; 2 = investment with medium risk, medium risk, medium return, and medium loss; 3 = investment with high risk, high risk, high return, and high loss)	1.279	0.544

**Table 3:** Correlation between financial literacy and rural households’ entrepreneurship.

	Model 1	Model 2	Model 3	Model 4
	Probit	Probit	IV-Probit	IV-Probit
Financial literacy	0.346 ***	0.173 ***	1.026 ***	0.746 **
	(0.054)	(0.063)	(0.211)	(0.320)
Age of household head		−0.015 ***		−0.012 ***
		(0.004)		(0.004)
Gender of household head		0.120		0.172
		(0.153)		(0.158)
The health level of household head		0.119 ***		0.105 ***
		(0.040)		(0.041)
Education level of household head		−0.007		−0.022
		(0.012)		(0.015)
Risk perception		−0.029		−0.043
		(0.027)		(0.028)
Number of families		0.075 ***		0.065 ***
		(0.023)		(0.024)
Cadre		0.060		−0.002
		(0.107)		(0.114)
party member		−0.019		−0.127
		(0.091)		(0.110)
Number of entrepreneurs among relatives		0.135 ***		0.124 ***
		(0.017)		(0.019)
Entrepreneurship failure experience		1.215 ***		1.091 ***
		(0.224)		(0.240)
Distance from the village to the nearest bank		0.024 *		0.027 **
		(0.013)		(0.013)
Entrepreneurship training		−0.014		−0.017
		(0.029)		(0.029)
Financial lectures		−0.040		−0.024
		(0.034)		(0.036)
Financial literacy at the village level			0.819 ***	0.600 ***
			(0.057)	(0.056)
Constant	−1.238 ***	−1.128 ***	−1.245 ***	−1.147 ***
	(0.036)	(0.399)	(0.037)	(0.406)
LRχ2/Wald χ2	40.583 ***	219.150 ***	23.640 ***	174.479 ***
First stage F value	/	/	207.70	39.01
endogenous Wald χ2	/	/	11.92 ***	3.46 *
N	2278	2278	2278	2278

Note: Robust standard errors in parentheses; * *p* < 0.1, ** *p* < 0.05, *** *p* < 0.01.

**Table 4:** Test of the mediating mechanism of credit constraint.

	Path 1: Ease Traditional Credit Constraints			Path 2: Ease Digital Credit Constraints		Path 3: Ease Online Credit Constraints	
	Model 5	Model 6	Model 7	Model 8	Model 9	Model 10	Model 11
	Entrepreneur	TCC	Entrepreneur	DCC	Entrepreneur	OCC	Entrepreneur
FL	0.173 ***	−0.370 ***	0.149 **	−0.632 ***	0.168 ***	−0.452 ***	0.168 ***
	(0.064)	(0.076)	(0.065)	(0.121)	(0.065)	(0.096)	(0.065)
TCC			−0.466 ***				
			(0.154)				
DCC					−0.301		
					(0.448)		
OCC							−0.164
							(0.249)
Controls	YES	YES	YES	YES	YES	YES	YES
Wald χ2	146.414 ***	136.305 ***	157.059 ***	93.708 ***	146.839 ***	87.285 ***	148.005 ***
Pseudo R2	0.137	0.138	0.143	0.256	0.137	0.153	0.137
N	2278	2278	2278	2107	2278	2278	2278

Note: Robust standard errors in parentheses; ** *p* < 0.05, *** *p* < 0.01. Because the first step test of Path 2 and Path 3 is consistent with the results of Model 5, the result is not repeated. To beautify the table, here financial literacy is abbreviated FL, traditional credit constraint is abbreviated TCC, digital credit constraint is abbreviated DCC, and online credit constraint is abbreviated OCC.

**Table 5:** Moderating mechanism test of risk preference.

	Entrepreneurship	
	Model 13	Model 14
Financial literacy	/	0.745 **
	/	(0.311)
Risk preference	0.294 ***	0.252 ***
	(0.062)	(0.071)
Risk preference * Financial literacy	/	−0.212 *
	/	(0.114)
Controls	YES	YES
Constant	−1.617 ***	−1.235 ***
	(0.397)	(0.419)
Wald χ2	166.86 ***	189.970 ***
First stage F value	/	40.04
endogenous Wald χ2	/	3.75 *
N	2187	2187

Note: Standard errors in parentheses; * *p* < 0.1, ** *p* < 0.05, *** *p* < 0.01.

**Table 6:** Robustness Test Results.

	CMP	IV-Probit
Financial literacy	0.568 ***	
	(0.209)	
New financial literacy		0.489 **
		(0.213)
Constant	−1.057 ***	−1.480 ***
	(0.392)	(0.439)
Financial literacy at the village level	0.819 ***	0.907 ***
	(0.057)	(0.106)
atanhrho_12/ F value	−0.242 *	31.45
	(0.129)	
LRχ2/Wald χ2	381.928 ***	171.234 ***
Endogenous Wald χ^2^	/	3.46 *
N	2278	2278

Note: Robust standard errors in parentheses; * *p* < 0.1, ** *p* < 0.05, *** *p* < 0.01.

## Data Availability

The data for this study are from the China Land Economy Survey (CLES) 2021 by Nanjing Agricultural University. Detailed information can be found at http://jscv.njau.edu.cn/#/index (accessed on 1 March 2023). Codes are unavailable due to privacy or ethical restrictions.
